# Cellular Model of Endotoxin Tolerance in Astrocytes: Role of Interleukin 10 and Oxylipins

**DOI:** 10.3390/cells8121553

**Published:** 2019-12-01

**Authors:** Dmitry V. Chistyakov, Alina A. Astakhova, Nadezda V. Azbukina, Sergei V. Goriainov, Viktor V. Chistyakov, Marina G. Sergeeva

**Affiliations:** 1 Belozersky Institute of Physico-Chemical Biology, Lomonosov Moscow State University, Moscow 119992, Russia; alina.an.astakhova@gmail.com (A.A.A.); mg.sergeeva@gmail.com (M.G.S.); 2 Faculty of Bioengineering and Bioinformatics, Lomonosov Moscow State University, Moscow 119234, Russia; ridernadya@gmail.com; 3 SREC PFUR Peoples’ Friendship University of Russia (RUDN University), Moscow 117198, Russia; goryainovs@list.ru (S.V.G.); chistvic@gmail.com (V.V.C.)

**Keywords:** astrocytes, inflammation, endotoxin tolerance, eicosanoids, oxylipins, interleukin-10

## Abstract

A phenomenon of endotoxin tolerance where prior exposure of cells to minute amounts of lipopolysaccharide (LPS) causes them to become refractory to a subsequent high-amount endotoxin challenge is well described for innate immune cells such as monocytes/macrophages, but it is still obscure for brain cells. We exposed primary rat cortical astrocytes to a long-term low-grade concentration of LPS, followed by stimulation with a middle-grade concentration of LPS. Inflammatory markers, i.e., pro-inflammatory cytokine TNFα, inducible enzymes COX-2 and iNOS, anti-inflammatory cytokine interleukin 10 (IL-10) detected at the mRNA and protein levels reveal similarities between astrocytes and macrophages in the model, i.e., tolerance in pro-inflammatory markers and priming in IL-10. Long-term or short-term treatment with IL-10 does not change cell sensitivity for LPS, which makes doubtful its involvement in the mechanisms of cell tolerance development. Significant changes occur in the oxylipin profiles measured by UPLC-MS/MS analysis. The priming occurs in the following compounds: 11-HETE, PGD_2_, PGE_2_, cyclopentenone prostaglandins, and TXB_2_. Tolerance is observed for 12-HHT, PGF_2α_, and 6-keto-PGF_1α_. As far as we know, this is the first report on changes in oxylipin profiles in the endotoxin tolerance model. The data can greatly improve the understanding of oxylipins’ role in inflammatory and resolution processes in the brain and mechanisms of astrocyte involvement in neuroinflammation.

## 1. Introduction

At present, the concept of inflammation is being redefined. Previously, it was viewed as a complex pathophysiological state, adopted primarily by innate immune cells in response to infection or tissue damage [1,2]. An appreciation of both inflammation as a self-regulating process and the concept of the resolution of inflammation as an active process has recently developed [1,3]. In addition, it is now generally accepted that, besides genetics affecting the risk posed by many chronic diseases, for instance, neurodegenerative diseases, there is a strong non-genetic contribution to the risk, which is poorly understood [2]. Among such non-genetic contributors, the modulation of innate immune system by endotoxin penetration and subsequent abnormality in innate immune system responses, i.e., inflammatory responses, have been the focus of recent investigations [2]. This prompts questions concerning the endotoxin tolerance phenomenon. It is known that sublethal doses of Gram-negative bacteria and their associated endotoxins, primarily, lipopolysaccharide (LPS), and protect animals from a lethal injection of endotoxin [4]. At the cellular level, the phenomenon has been described as a type of tolerance in which exposure to low concentrations of endotoxins reprograms cells, with responses to further endotoxin challenges compromised. Although the molecular mechanisms underlying endotoxin tolerance remain elusive [2,4], some features are described for macrophages [4]. In classic immune cells, recurrent stimulations with LPS induce a specific state in which cells decrease (“tolerate”) production of pro-inflammatory mediators (IL-6 (interleukin 6), TNFα (tumor necrosis factor alpha), iNOS (inducible nitric oxide synthase), and COX-2 (cyclooxygenase 2) and others) but instigate (“sensitize”) the synthesis of anti-inflammatory mediators (IL-10 (interleukin 10) and others). This condition is referred to as a protective mechanism, targeted at the prevention of excessive toxic damage from cytokine production [2].

Neuroinflammation is a form of innate immune response initiated by altered homeostasis within brain tissues. Neuroinflammation underlies the pathology of neurological disorders, such as multiple sclerosis, and contributes to other neurodegenerative diseases, including Alzheimer’s disease, Parkinson’s disease and amyotrophic lateral sclerosis, as well as other brain pathologies, including post-ischemic neurodegeneration, and traumatic, metabolic, toxic, and neoplastic disturbances [5,6,7]. Astrocytes are immunocompetent cells of the nervous tissue which play an essential role in neuroinflammation [5,6,8,9], sense LPS signals and promptly respond to pro-inflammatory challenges by activation of downstream signaling cascades, including conventional markers of inflammation: IL-6, TNFα, COX-2 etc. [6,10,11,12,13,14]. Abnormal inflammation-related activation of astrocytes is considered with regard to major pathophysiological forces driving CNS disorders, including Alzheimer’s disease, Parkinson’s disease, and traumatic injuries of the brain [9]. In addition to typical pro-inflammatory and neurotoxic genes, part of the gene expression program may be devoted to the resolution of inflammation, with gene products offering context-dependent pro-inflammatory or pro-resolving functions [1,15]. A prominent example is the inducible cyclooxygenase 2 (COX-2), a key enzyme of eicosanoid biosynthesis. Its products, i.e., prostaglandins, show both pro-inflammatory and pro-resolving qualities [5,15,16]. Our recent studies revealed the complex regulation of COX-2 expression both at the transcriptional and post-transcriptional levels in primary rat astrocytes [12,13,14]. All these data indicate the importance of characterizing the synthesis of prostaglandins and other oxylipines for understanding the mechanisms of astrocyte responses to inflammatory stimuli. The phenomenon of endotoxin tolerance in the case of astrocytes has not been practically studied. Data on repeated stimulations with different concentrations of LPS indicate that these cells can change their sensitivity to repeated stimulations [17,18,19]. In the works of Beurel et al., middle-grade concentrations of LPS (100 ng/mL) were added to murine astrocytes for 24 h and then stimulated with a low-grade concentration of LPS (10 ng/mL) for 24 h [17,18]. This is a completely different model, since treatment (100 ng/mL LPS, 24 h) is used to create a pro-inflammatory phenotype of astrocytes [20], while endotoxin tolerance effects concerning sustainability of low concentrations treated cells for further endotoxin attack. An important question in characterizing inflammatory responses in astrocytes is their interaction with partners (microglia, neurons, neighboring naive astrocytes). When studying the LPS effects on brain cells, the focus of attention is on the interactions between astrocytes and microglia or astrocytes and neurons [6,20,21,22]. At the same time, information about the possibility of the stimulated astrocytes effects on naive astrocytes remains scarce. Therefore, we exposed astrocytes to a long-term low-grade concentration of LPS (10 ng/mL, 48 h) and investigated their sensitivity, followed by stimulation with a middle-grade concentration of LPS (100 ng/mL, 4 h). We characterized inflammatory markers, cytokine TNFα, inducible enzymes COX-2 and inducible nitric oxide synthase (iNOS) and anti-inflammatory cytokine interleukin 10 (IL-10), and, then estimated the oxylipins profiles in the model. We showed that astrocytes being exposed to low concentrations of LPS, release factors into the extracellular environment, which can affect the sensitivity of naive astrocytes to LPS. Our data show that IL-10 is unlikely could perform this function, while an involvement of oxylipins is possible.

## 2. Materials and Methods

### 2.1. Reagents

Lipopolysaccharide (LPS) (Sigma-Aldrich, cat.no L2630 St. Louis, MO, USA), streptomycin–penicillin (cat.no A063), trypsin (cat.no P037), EDTA, fetal bovine serum (cat.no BS-110/500) were from PanEco (Moscow, Russia). Culture medium Dulbecco’s Modified Eagle Medium (DMEM) (cat.no 21885-025) (Gibco, Thermo Fisher Scientific, Waltham, MA, USA). Antibodies against COX-2 (D5H5, cat.no 12282) and β-tubulin (Sigma Chemicals, Taufkirchen, Germany), secondary horseradish peroxidase conjugated antibodies (anti-rabbit, anti-mouse, and anti-goat) (SCBT and CST), western blotting substrate ECL (Thermo Fisher Scientific, cat.no 32209, Waltham, MA, USA). Oasis^®^ PRIME HLB cartridge (60 mg, 3cc, cat.no. 186008056) were obtained from Waters, Eschborn, Germany.

### 2.2. Primary Cell Culture

The cells were obtained from one- or two-day old pups of Wistar rats. All of the experimental procedures were performed according to the guidelines in the European Convention for the Protection of Vertebrate Animals used for Experimental and Other Scientific Purposes. The cultures of primary rat astrocytes were obtained from newborn rats of both sexes, as previously reported [12]. In brief, the brains from decapitated pups were rinsed with ice-cold Puck’s solution (137.0 mM NaCl, 5.4 mM KCl, 0.44 mM KH2PO4, 0.3 mM Na2HPO4, and 5.5 mM glucose, pH 7.4) and triturated against nylon meshes with the pores of 250 and 136 μm, in a consecutive order. The dissociated cells were plated into 75 cm2 culture flasks at a density of 6 × 105 cells per mL. The cells were subsequently cultured in DMEM (1 g/L D-glucose, 10% bovine fetal serum [FBS], 50 units/mL streptomycin, 50 μg/mL penicillin) at 37 °C, with 10% CO2. After five days of cultivation in DMEM, the culture medium was replaced with a fresh medium and the flasks were placed on a shaker at 200 rpm for 4 h to dissociate the microglial cells. The microglia containing medium was discarded and the astrocytes-enriched cultures were further grown for the following four days, and the medium was replaced every two days. Subsequently, the cells were washed with phosphate buffered saline and detached from the plastic with trypsin–EGTA solution and plated into six-well plates, and were maintained for two days in DMEM. After this, the medium was replaced by the medium of the same composition, and the cells were used for the experiments.

### 2.3. Measurement of the Relative RNA Expression Level

Total mRNA was isolated using the GeneJET RNA Purification Kit (Thermo Scientific, Waltham, MA, USA). The concentration of RNA was measured using an Implen NanoPhotometer C. cDNA was generated according to the manufacturer’s instructions using the MMLV RT kit (Evrogen, Moscow, Russia) with oligo-(dT)-primers. Real-time PCR was performed using the 5x PCR-HS-SYBR mix (Evrogen, Moscow, Russia) and the DTlite 4 amplificator (DNATechnology, Moscow, Russia). The sequences of PCR primers used in this study were as follows: β-actin: forward 5′-TCATCACTATCGGCAATGAGCGGT-3′, reverse 5′ACAGCACTGTGTTGGCATAGAGGT3′; TNFα: forward 5′-CAAGGAGGAGAAGTTCCCAA-3′ reverse 5′-TGATCTGAGTGTGAGGGTCTG-3′; IL-10: forward 5′-CCCAGAAATCAAGGAGCATTTG-3′, reverse 5′-TCATTCTTCACCTGCTCCAC-3′; COX-2 forward 5′-TGTACAAGCAGTGGCAAAGG-3′, reverse 5′-TAGCATCTGGACGAGGCTTT-3′, iNOS forward 5′-CCACAATAGTACAATACTACTTGG-3′, reverse 5′-ACGAGGTGTTCAGCGTGCTCCACG-3′ the annealing temperature was 57 °C. Expression of each gene was measured in 25 µL reactions using cDNA synthesized from 70 ng RNA per reaction well. The relative mRNA expression level was determined by the Δ*C*T method. The β-actin gene was used as a constitutive gene for normalization. The level of normalized gene expression in control cells or in stimulated cells (specified directly in the text) was taken as one.

### 2.4. Western Blot Analysis

Cultured cells were lysed in RIPA buffer (Sigma) containing cocktails of protease (cat.no. 5892791001, Roche) and phosphatase (cat.no. 4906845001, Roche) inhibitors. Then, the protein concentration was measured using DC Protein Assay Kit (Bio-Rad, Hercules, CA, USA). Primary antibodies concentration to the following proteins were used: Anti-COX-2 (1:2000) at 4 °C overnight.; and secondary horseradish peroxidase conjugated antibodies (anti-rabbit, anti-mouse) (SCBT, CST). Membranes were developed using SuperSignal West Femto Maximum Sensitivity Substrate or SuperSignal West Pico Chemiluminescent Substrate (Thermo Scientific, MA, USA). Luminescence was detected by means of ChemiDoc XRS+ system (Bio-Rad), and the luminescence intensity was calculated with Image Lab 3.0 software (Bio-Rad).

### 2.5. UPLC-MS/MS Conditions and Sample Preparation

After the experiments, the supernatant was collected and stored at −80 °C for further analysis. The cell-free culture media were taken for the solid-phase lipid extraction (Oasis^®^PRIME HLB cartridge (60 mg, 3cc)) as described before [23]. For identification of lipid mediators, the respective lipid extracts were analyzed using 8040 series UPLC-MS/MS mass spectrometer (Shimadzu, Japan) in multiple-reaction monitoring mode at a unit mass resolution for both the precursor and product ions [23]. Comprehensive analysis of lipid metabolites was performed by using a composition of internal standards (tetranor-PGEM-d6 (cat.no. 314840), 6-keto PGF_1α_-d4 (cat.no. 315210), TXB_2_-d4 (cat.no. 319030), PGF_2α_-d4 (cat.no. 316010), PGE_2_-d4 (cat.no. 314010), PGD_2_-d4 (cat.no. 312010), Leukotriene (LT) C_4_-d5 (cat.no. 10006198), LTB_4_-d4 (cat.no. 320110), 5(S)-HETE-d8 (cat.no. 334230), 12(S)-HETE-d8 (cat.no. 334570), 15(S)-HETE-d8 (cat.no. 334720), PAF C16-d4 (cat.no. 10010229), Oleoyl Ethanolamide-d4 (cat.no. 9000552), PGA_2_-d4 (cat.no. 310210) (Cayman Chemical, Ann Arbor, MI, USA) and a commercial software method package for lipid mediators (Lipid Mediator Version 2 software package Shimadzu, Kyoto, Japan) according to the manufacturer’s instructions. The concentration of lipids was normalized to the total protein and was expressed as pg/mg. The total protein was determined by the Bradford assay.

### 2.6. Determination of TNFα and IL-10 by Enzyme-Linked Immunoassay

After the experiments, the supernatant was collected and stored at −70 °C for the further analysis. The levels of the released TNFα and IL-10 were determined using an enzyme-linked immunoassay commercial kits and Synergy H4 plate reader (BioTek, Winooski, VT, USA), following the manufacturer’s instructions.

### 2.7. Experimental Data Analysis and Statistics

The data are expressed as mean ± SEM. The normality of data sets was assessed using the Shapiro–Wilk test. The data were subjected to a one-way ANOVA, followed by Bonferroni’s post hoc test, in order to determine the statistical significance. *p* < 0.05 was considered statistically significant. All of the experiments were repeated at least three times.

## 3. Results

**Figure 1:**
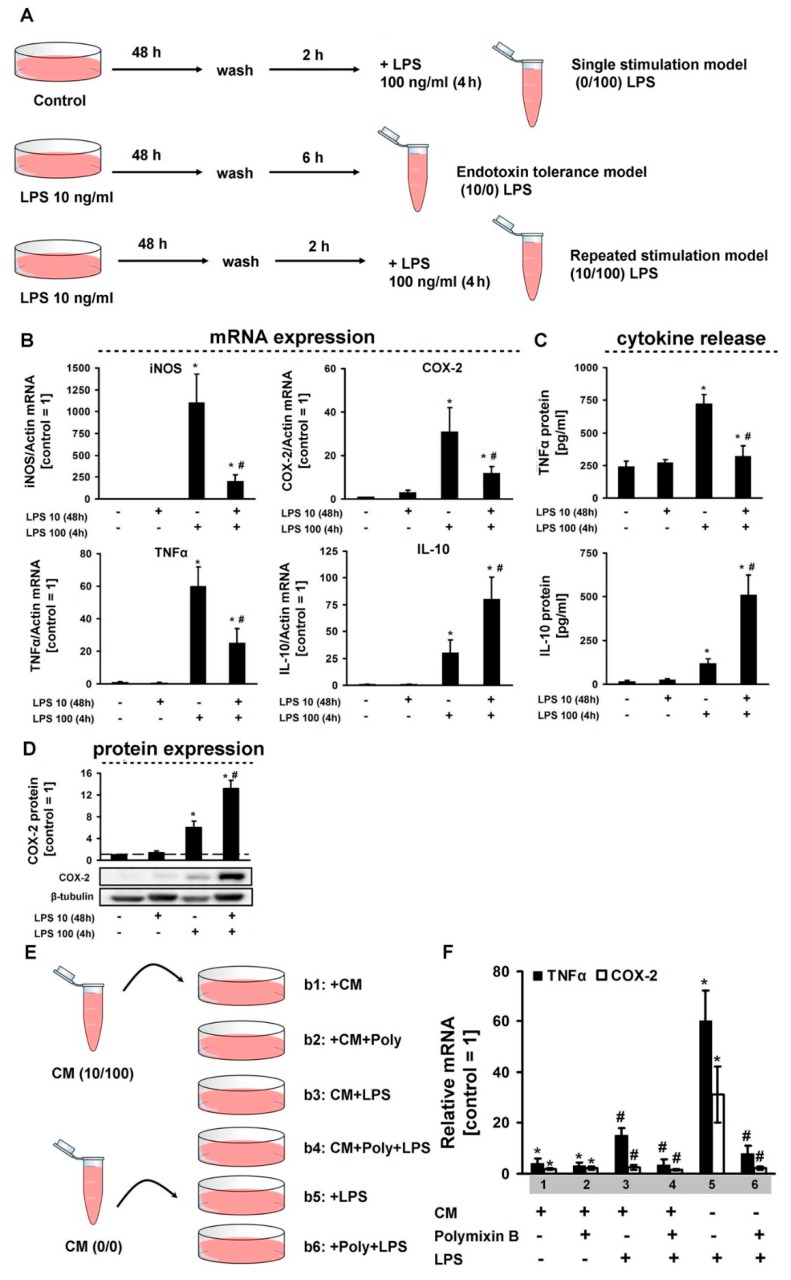
Changes in pro- and anti-inflammatory markers in the cell model of endotoxin tolerance. (**A**)—a general scheme of stimulations. Astrocytes were stimulated with lipopolysaccharide (LPS) (100 ng/mL) for 4 h (0/100 LPS), or astrocytes were grown in media LPS (10 ng/mL) for 46 h, washed and retained in fresh media for additional 2 h (10/0) and then stimulated with LPS (100 ng/mL) for 4 h (10/100). (**B**)—mRNA expression of indicated genes in astrocytes treated with LPS (10 ng/mL and 100 ng/mL) for 48 h and 4 h, respectively. Values are normalized to β-actin mRNA levels. Results are expressed as fold-changes, relative to untreated cells. (**C**)—TNFα and IL-10 protein release measured by ELISA in supernatant samples. (**D**)—western analysis of COX-2 expression. (**E**)—scheme of condition medium (CM) treatment. Astrocytes were grown in CM medium, diluted 1:1 with fresh DMEM and then stimulated with LPS (100 ng/mL) alone or in combination with polymyxin B (Poly, 50 μg/mL). (**F**)—mRNA expression of indicated genes. Values are normalized to β-actin mRNA levels and results are expressed as fold-changes, relative to untreated cells. Values represent mean ± SEM from three independent experiments. * *p* < 0.05, compared with unstimulated cells, # *p* < 0.05, compared with the LPS-stimulated cells.

Whereas the ability of astrocytes to respond to LPS with increased mRNA expression and protein release of various pro-inflammatory genes is well documented, the question as to how endotoxin tolerance affects these responses has not been addressed so far. For our analysis, we used a model of primary rat cortical astrocytes exposed to a low-grade concentration of LPS (10 ng/mL for 48 h), followed by stimulation with a middle-grade concentration of LPS (100 ng/mL for 4 h) (Figure 1A). We then evaluated, at the mRNA and the protein level, the pro-inflammatory (TNFα) and anti-inflammatory (IL-10) cytokines (Figure 1B–D). We discovered that, a low-grade concentration of LPS does not influence cytokines or enzymes expressions at the mRNA level (Figure 1B), not influence at levels of TNFα or IL-10 release in extracellular medium (Figure 1C), not influence the intracellular protein level (Figure 1D). There is a tolerance towards TNFα, iNOS, COX-2 and priming for IL-10 at the mRNA levels for secondary stimulation (Figure 1B). Changes at the mRNA level are accompanied by similar changes in the levels of cytokines released into the intercellular medium (Figure 1C). For COX-2 intracellular expression we obtained priming at the protein level (Figure 1D). To assess the possibility of cells in the endotoxin tolerance model being able to modulate the responses of naive cells, we used the processing scheme shown in Figure 1E. Medium from the cellular cultures in the endotoxin tolerance model was selected, and LPS was blocked by polymyxin and added to the naive cells. The sensitivity of these cells for LPS stimulation was evaluated by TNFα and COX-2 mRNA expression (Figure 1F). We discovered that the cell environment in the endotoxin tolerance model makes other cells insensitive to LPS (Figure 1F). This allows to suppose some components in medium of treated cells, which may modulate sensitivity of naive cells.

**Figure 2:**
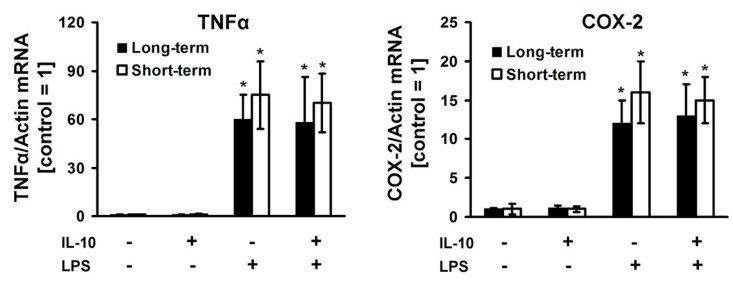
The effect of short-term and long-term interleukin-10 incubation on the LPS-mediated response in astrocytes. Astrocytes were pretreated with interleukin-10 (IL-10, 20 ng/mL) for 1h (short-term, white columns) or 24 h (long-term, black columns) and then stimulated with LPS (100 ng/mL) for 4 h. The mRNA levels were determined by real-time RT-PCR. Values are normalized to β-actin mRNA levels. Results are expressed as fold-changes, relative to untreated cells. Values represent mean ± SEM from three independent experiments. * *p* < 0.05, compared with the unstimulated cells.

Cytokine IL-10 is associated with the development of anti-inflammatory processes in the brain [24]. A significant increase in the anti-inflammatory cytokine IL-10 level in the endotoxin tolerance model (Figure 1C) suggested the possibility of its involvement in mechanisms of cell tolerance in condition medium experiments (Figure 1F). Therefore, we tested the expression of TNFα and COX-2 mRNA during short-term (1 h) and long-term (24 h) IL-10 treatment before LPS stimulation (Figure 2). LPS causes an increase in the expression of TNFα (more than 60-fold) and COX-2 (more than 12-fold). In relation to the added IL-10, in both time-term expositions we obtained no difference in cell responses to LPS.

**Figure 3:**
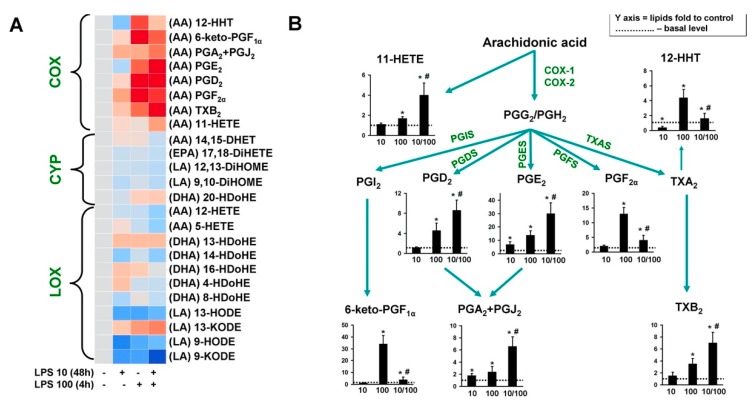
Oxylipins release in the cellular model of endotoxin tolerance. (**A**) Heat map representation of oxylipin production of n-6 and n-3 fatty acid-derived lipid mediators. Astrocytes were treated with LPS (10 ng/mL) for 48 h, then the cells were washed and stimulated with LPS (100 ng/mL) for 4 h. Concentrations of oxylipins in supernatants were measured using UPLC-MS/MS. The heat map shows relative amounts of each lipid mediator compared to the control. The horizontal axis indicates the stimuli, while the vertical axis indicates the relative amount (log2) of each lipid mediator. Metabolites were divided into: Lipoxygenase (LOX), cyclooxygenase (COX), and cytochrome (CYP) pathways involved in their synthesis. (**B**) Comparative eicosanoid profile in astrocytes stimulated with LPS 10 ng/mL for 48 h alone or in combination with LPS 100 ng/mL for 4h. * *p* < 0.05, compared with the unstimulated cells, # *p* < 0.05, compared with the LPS-stimulated cells.

Oxylipins can play a modulating role that affects the ability of astrocytes to change their sensitivity to LPS stimulation [16,25]. Therefore, we studied the oxylipin synthesis in our endotoxin tolerance model. Astrocytes were treated with 10 ng/mL of LPS for 48 h, 100 ng/mL of LPS for 4 h, or both (LPS 10 + LPS 100) (Figure 3). All LPS treatments had almost the same effect on the oxylipins formed during the lipoxygenase or epoxygenase oxidation pathways for all substrates (arachidonic, docosahexaenoic, linoleic acids) (Figure 3A). Significant changes occurred in the oxylipin metabolism via the cyclooxygenase pathway (Figure 3B). The amount of oxylipins in the extracellular medium changed by a factor of 2:3 (Figure 3B). The priming occurred in the following compounds: 11-hydroxyeicosatetraenoic acid (11-HETE), prostaglandins D2 (PGD_2_), and E2 (PGE_2_), cyclopentenone prostaglandins (PGA_2_ + PGJ_2_), thromboxane B2 (TXB_2_). Tolerance was observed in the case of 12-hydroxyheptadecatrienoic acid (12-HHT), prostaglandin F_2α_ (PGF_2α_) and 6-keto-prostaglandin F_1α_ (6-keto-PGF_1α_). The synthesis of these substances was practically stopped.

## 4. Discussion

We found that astrocytes share similar features with macrophages in the endotoxin tolerance cellular model, i.e., tolerance in the case of pro-inflammatory genes and priming for the expression and release of the anti-inflammatory cytokine IL-10 were observed. It is noteworthy that astrocytes are cells of ectodermal origin, while macrophages as well as microglia are cells of hematopoietic origin. Moreover, it was previously shown that the activation of TLR4 signaling in astrocytes differs from that in myeloid cells in several major regulatory respects [26,27]. Thus, an important outcome of our studies on the molecular mechanisms of the endotoxin tolerance model for astrocytes was that an inflammatory response is not a specialized function of cells of hematopoietic origin, but rather a fundamental attribute of other cellular types. The observed similarity between endotoxin tolerance mechanisms reflects some general processes of adaptation to innate immunity, likewise in cells of immune and non-immune origin.

Several studies have indicated that activated astrocytes release factors that are neuroprotective [6] or can influence CNS immunity and provide negative feedback to activated microglia [21,22]. We demonstrated that changes in endotoxin tolerance affect not only the cells themselves, which are subjected to prolonged exposure to low concentrations of endotoxin, but also substances released into the medium in the endotoxin tolerance model, which can influence naive cell responses. It would seems that the most likely carrier of this information is the IL-10 The most likely carrier of this information is IL-10, since it was shown that this is an anti-inflammatory cytokine which has immunoregulatory effects in the brain [28]. Activated microglia [29] or astrocytes [12,30] synthesize and release IL-10 in response to pro-inflammatory stimuli. It was previously shown that IL-10 lowered the pro-inflammatory profile of LPS-activated astrocytes when added 1 h after stimulation [21]. On the contrary, our data show that treatment with IL-10 (1 h or 24 h), before the stimulus was added, did not affect the expression of inflammatory markers. This is an interesting phenomenon. The molecular mechanisms of this dependence of the TLR4 signaling pathway on the time of IL-10 addition require further investigation. Possibly, the sequence of signaling pathways activation is important, i.e., added IL-10 to the TLR-activated or LPS to IL-10 activated pathways. Indeed, although the expression of IL-10 and IL-10 receptor (IL-10R1) on astrocytes is well shown [31], most studies added IL-10 simultaneously or after LPS stimulation [21,31]. It was shown that IL-10 secretion is time-dependently induced by LPS, whereas IL-10R1 is constitutively expressed in astrocytes and down-regulated by IL-10 in LPS-treated cultures, therefore feedback mechanism for IL-10 was suggested [31]. From our data, it can be assumed that some other intracellular participants are required for the IL-10 effects manifestation in the LPS-induced response of astrocytes. In any case, our data indicate that IL-10 is unlikely to be an agent that induces the tolerance of naive cells when extracellular medium is added from cells treated in the endotoxin tolerance model.

Oxylipins may be other suspected substances for transmitting information from tolerant cells to naive ones. This class of substances includes both pro-inflammatory compounds and resolution substances, which are responsible for restoring the system after the pro-inflammatory stimulus has been applied [1,5]. Although COX-2 expression was used as a classic marker of inflammation for a long time, oxylipins were typically evaluated in one or two compounds. However, in astrocytes, LPS modulates the synthesis of a wide range of compounds that can affect the surrounding cells. Prostaglandins exert cellular affects through their specific receptors: The PGE_2_ receptors EP1, EP2, EP3 and EP4; the PGD_2_ receptors DP1 and DP2; the PGF_2α_ receptor FP; the PGI_2_ receptor IP; and the TXA_2_ receptor TP (most of them are found in astrocytes) [16]. The receptors signal, either via Gαs (EP2, EP4, IP, and DP1 receptors) or Gαs-proteins (EP3, DP2 and TP receptors), an increase in the intracellular levels of cyclic-adenosine monophosphate (cAMP) or an increase or decrease in the intracellular levels of cAMP and calcium, respectively. In addition, some substances could be agonists of transcription factors (i.e., PPARs) [32]. Some of these effects can be of an opposing nature, and some can be summed up [26]. It is obvious that the experimental paradigm should be moved from testing one substance towards the characterization of the influence of oxylipin mixtures. The effects are unknown at present because the testing of oxylipin profiles has only recently been started. Our data on oxylipin profiles in the cellular model of endotoxin tolerance in astrocytes show that priming occurs in the following compounds: 11-HETE, PGD_2_, PGE_2_, cyclopentenone prostaglandins (PGA_2_ and PGJ_2_), TXB_2_. Tolerance is observed for 12-HHT, PGF_2α_ and 6-keto-PGF_1α_. Interesting to note, that recently we have shown that mitochondrial inhibitors (rotenone, oligomycin, antimycin) modulate IL-1-triggered secretion of eicosanoids such as prostaglandin E2, prostaglandin F2, and 6-keto-prostaglandin F1α [14]. Since all these oxylipins have receptors in astrocytes, microglia, and neurons [16], such significant changes in oxylipin amounts can lead to significant changes in the responses of the surrounding cells. As far as we know, this is the first report concerning changes in the oxylipin ratio in the endotoxin tolerance model. Whether processes in mitochondria can take part in the manifestation of the endotoxin tolerance phenomenon is an intriguing challenge for further research. The data show that the oxylipin spectrum is obviously changing significantly. This should lead to changes in the responses of the entire cell population. As far as we know, this is the first report on changes to oxylipin profiles in the endotoxin tolerance model. The data can greatly improve the understanding of oxylipins’ role in inflammatory and resolution processes in the brain and mechanisms of astrocyte involvement in neuroinflammation.
